# Pattern of reading eye movements during monovision contact lens wear in presbyopes

**DOI:** 10.1038/s41598-018-33934-6

**Published:** 2018-10-22

**Authors:** Fabrizio Zeri, Shehzad A. Naroo, Pierluigi Zoccolotti, Maria De Luca

**Affiliations:** 1 Ophthalmic Research Group. School of Life and Health Sciences, Aston University, Birmingham, B4 7ET United Kingdom; 2 University of Milano Bicocca, Department of Materials Science, 20125 Milan, Italy; 3 Neuropsychology Unit, IRCCS Fondazione Santa Lucia, Rome, 00179 Italy; 4 Department of Psychology, Sapienza University, Rome, 00176 Italy

## Abstract

Monovision can be used as a method to correct presbyopia with contact lenses (CL) but its effect on reading behavior is still poorly understood. In this study eye movements (EM) were recorded in fifteen presbyopic participants, naïve to monovision, whilst they read arrays of words, non-words, and text passages to assess whether monovision affected their reading. Three conditions were compared, using daily disposable CLs: baseline (near correction in both eyes), conventional monovision (distance correction in the dominant eye, near correction in the non-dominant eye), and crossed monovision (the reversal of conventional monovision). Behavioral measures (reading speed and accuracy) and EM parameters (single fixation duration, number of fixations, dwell time per item, percentage of regressions, and percentage of skipped items) were analyzed. When reading passages, no differences in behavioral and EM measures were seen in any comparison of the three conditions. The number of fixations and dwell time significantly increased for both monovision and crossed monovision with respect to baseline only with word and non-word arrays. It appears that monovision did not appreciably alter visual processing when reading meaningful texts but some limited stress of the EM pattern was observed only with arrays of unrelated or meaningless items under monovision, which require the reader to have more in-depth controlled visual processing.

## Introduction

It has been estimated that presbyopia will affect about 2 billion people by 2050^1^. Spectacles are the oldest and widespread method of presbyopia correction^2^ and contact lenses (CL) and refractive surgery offer alternative solutions. Presbyopia correction with monovision CLs has been used since the sixties^3^; later, monovision was also applied in refractive surgery^4,5^. A recent international survey reported that 22% of people who were prescribed CLs to correct presbyopia (in order to avoid the use of reading glasses) were fitted with monovision CLs^6^.

In monovision one eye is corrected for distance (usually the dominant eye) and the other for near vision^3,4,7^. In this way, both eyes contribute to vision and clear vision can be potentially achieved at all distances, even if one eye sees more clearly at near whilst the other at far distance.

The level of subjective success of monovision is generally high with percentages between 59% and 67% in adapted CL wearers, and between 80% and 96% in surgery patients^7^. Still, several issues have been reported with monovision since it is a form of induced anisometropia (i.e. the two eyes have a different refraction) that causes a superimposition of an in-focus image over a blurred image, thus implying a certain degree of suppression^8^. Furthermore, it induces an impairment of stereopsis^9–14^.

The stress induced by monovision may affect the visual analysis in daily activities by slowing down information processing but evidence to support this claim is limited. A study based on eye movement (EM) recordings reported that artificially-induced monocular blur in visually-normal persons with contact lenses did not affect the execution of primary saccades in bringing the gaze toward a target, but it affected the frequency of secondary saccades^15^. In studies using simulated driving performance comparing monovision with other presbyopic visual corrections, monovision did not alter EM parameters recorded^16,17^. Anyway, the reading performance is rarely considered in studies examining presbyopia correction, and it is measured only in terms of reading speed, such as the number of words per minute (WPM)^18,19^. It appears that there are no studies on the effect of monovision on reading behavior that measured fine-grained, objective indexes reflecting temporal and spatial details of the reading process, such as those provided by EM recordings.

The present study aims to examine the impact of monovision CL correction on reading at near distance through EM recorded while participants read text passages, as well as arrays of unrelated words and non-words (these latter are strings of letters that are used to create a readable item that actually is not a word and has no meaning). The reading of text passages allows evaluating the presbyopes’ performance in a condition comparable to day life reading situations. The arrays of unrelated items (either words or non-words) represent a sensitive test, more likely to be effective in detecting the effects of monovision in reading processing since they call for a fine visual examination of the orthographic materials: the arrays of unrelated words require more in-depth inspection of the text with respect to text passages (because a meaningful context is absent), while the arrays of non-words may in turn be more sensitive than the arrays of words in highlighting possible stressful effects of monovision since the reader cannot solve them either relying on the context or on the visual lexicon, but only through an in-depth serial item-by-item analysis.

This study is designed as a prospective experimental crossover study based on a repeated measure design, to examine the effect of a conventional monovision CL correction on reading in a group of healthy presbyopic participants who were naïve to monovision. A condition of conventional monovision CL correction was compared to a balanced CL correction for near vision (baseline condition from here on); in the conventional monovision condition, the dominant eye was corrected for far and the non-dominant eye for near vision; in the baseline condition, both eyes were fitted with single vision CLs with correction for near vision. Furthermore, a condition of crossed monovision (the dominant eye corrected for near, and the non-dominant for far vision which represent the reverse of conventional monovision) served as an additional condition. The effect of the three CL conditions on reading performance was evaluated through both behavioral and EM reading parameters.

## Results

### Preliminary visual assessment

An initial examination with standard optometric tests was performed to verify the eligibility of participants according to the inclusion criteria, to assess their basic visual and reading performance, to test their general performance in monovision, and to determine the power of the different CLs to be used for the three conditions of the EM recordings.

#### Basic visual and reading performance

Descriptive statistics on the basic performance of the presbyopic group are reported in Table 1 and in the first two columns of Table 2. Table 1 shows that the group was characterized by a consolidated presbyopia with low amplitude of accommodation and a significant level of addition required at near (that is the need for positive lens powers to compensate for presbyopia). The first two columns of Table 2 report reading performance at near (40 cm) when binocular correction either at distance or at near was used. Here, the group showed very poor reading performance at near when wearing subjective refraction at distance that is restored with near correction. As for eye dominance, eight participants had right eye and seven had left eye dominance. The mean subjective index of the impact of presbyopia measured by the Near Activity Vision Questionnaire (NAVQ) was 46 ± 15 (range 23/71) indicating low satisfaction when reading at near without any correction.

**Table 1:** Basic visual performance of participants with presbyopia. Mean and standard deviations (with ranges in parentheses) for the preliminary visual assessment variables (RE and LE indicate the right and left eye, respectively).

Mean Spherical Equivalent (D)	RE −0.78 ± 1.83 (+1.13/−4.75) LE −0.78 ± 1.80 (+1.13/−4.50)
Addition for near (D)	RE 1.55 ± 0.29 (1.25/2.00) LE 1.55 ± 0.29 (1.25/2.00)
Best Corrected Visual Acuity (logMAR)	RE −0.10 ± 0.07 (0.00/−0.20) LE −0.12 ± 0.08 (−0.02/−0.24)
Best Corrected Visual Acuity monocular difference (log MAR)	0.04 ± 0.03 (0.00/0.08)
Accommodation Amplitude (D)	RE 2.25 ± 0.57 (1.45/3.33) LE 2.27 ± 0.58 (1.45/3.33)
H pattern test	None of the subjects resulted positive
Horizontal heterophoria (Dissociated phoria; Δ)	at distance −0.6 ± 0.9 (1.0/−2.5) at near −2.4 ± 3.0 (2.0/−8.0)
Fusional Reserves (Δ)	Base Out blur at distance 11.3 ± 4.1 (7.0/20.0) Base Out break at distance 13.7 ± 4.5 (10.0/25.0) Base Out recovery at distance 10.8 ± 4.5 (6.0/20.0) Base In break at distance 6.7 ± 2.5 (4.0/12.0) Base In recovery at distance 4.1 ± 1.9 (2.0/8.0)
Near point of convergence (cm)	break 7.7 ± 2.4 (4.0/11.0) recovery 10.7 ± 2.9 (7.0/16.0)
Stereoacuity (arcsec)	30 ± 24 (10/80)
Fixation disparity (Associated phoria; ∆)	0.0 ± 0.7 (1.0/−2.0)
Central Suppression	0.6 ± 1.5 (0/5)

**Table 2:** First three columns: group means and SDs (with ranges in parentheses) for the binocular reading acuity and critical print size (CPS) in the Radner test in three conditions: subjective refraction at distance, correction at near (i.e., subjective refraction at distance plus the final addition at near), and monovision. Last three columns: results of the t-tests comparisons.

	Subjective refraction at distance	Correction at near	Monovision	Subjective refraction at distance vs. Correction at near	Subjective refraction at distance vs. Monovision	Correction at near vs. Monovision
Reading Acuity (logMAR)	0.21 ± 0.20 (0.61/−0.08)	−0.01 ± 0.09 (0.10/−0.18)	0.07 ± 0.11 (0.30/−0.10)	t_(14)_ = 4.3 p < 0.005	t_(14)_ = 3.3 p < 0.01	t_(14)_ = 3.7 p < 0.005
CPS (logMAR)	0.41 ± 0.18 (0.70/0.20)	0.18 ± 0.11 (0.40/0.00)	0.27 ± 0.13 (0.60/0.10)	t_(14)_ = 6.7 p < 0.001	t_(14)_ = 4.0 p < 0.005	t_(14)_ = 4.5 p < 0.001

#### Comparisons between basic visual performance and monovision correction

Statistical comparisons were run to evaluate the impact of monovision with respect to basic visual functioning. Best corrected visual acuity (BCVA) was recalculated for the dominant and non-dominant eye to offer a comparison with the monovision condition. A paired t-test was performed to compare the BCVA of the non-dominant eye with the visual acuity (VA) at distance of the non-dominant eye when corrected for near in monovision. These acuities were measured as the logarithm of the minimum angle of resolution (logMAR). A Wilcoxon Signed Rank test was used separately for stereoacuity threshold (measured in seconds of arc, arcsec) and the level of central suppression to evaluate the difference between the binocular near correction and monovision correction. Paired t-tests separately for reading acuity and critical print size (CPS) were performed to evaluate the differences among the conditions of subjective refraction at distance, the correction at near, and monovision (both the variables were measured in logMAR).

Monovision significantly changed some visual functions: in the non-dominant eye the VA at distance dropped significantly from −0.12 logMAR (for the subjective refraction at distance) to 0.32 logMAR (for monovision; t_(14)_ = −16.7, p < 0.0001); stereoacuity was impaired with threshold passing on average from 30 arcsec (for the balanced correction at near) to 155 arcsec (for monovision; p < 0.001); the level of central suppression increased from 0.6 (for the balanced correction at near) to 2.2 (for monovision; p < 0.05). As expected, reading acuity was higher and CPS was smaller with correction at near than with subjective refraction at distance (see Table 2); reading parameters were also better for monovision with respect to the subjective refraction at distance, but worse for monovision with respect to the correction at near.

### Eye movement and behavioral parameters for the reading of passages

**Figure 1:**
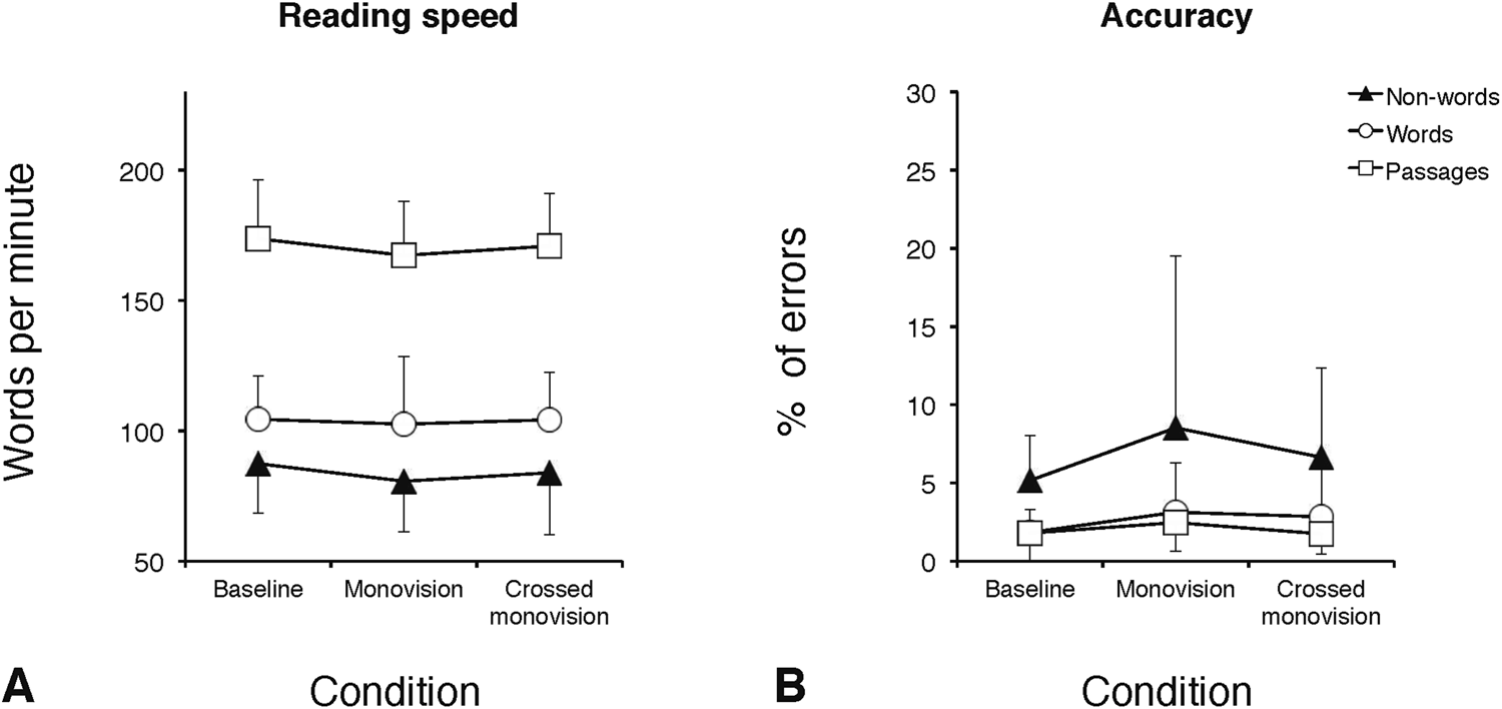
Group means for reading speed (in WPM) and accuracy (in percentage of errors) as a function of the three experimental CL conditions (baseline, monovision, and crossed monovision), separately for text passages, words, and non-words. Error bars represent standard deviations.

**Figure 2:**
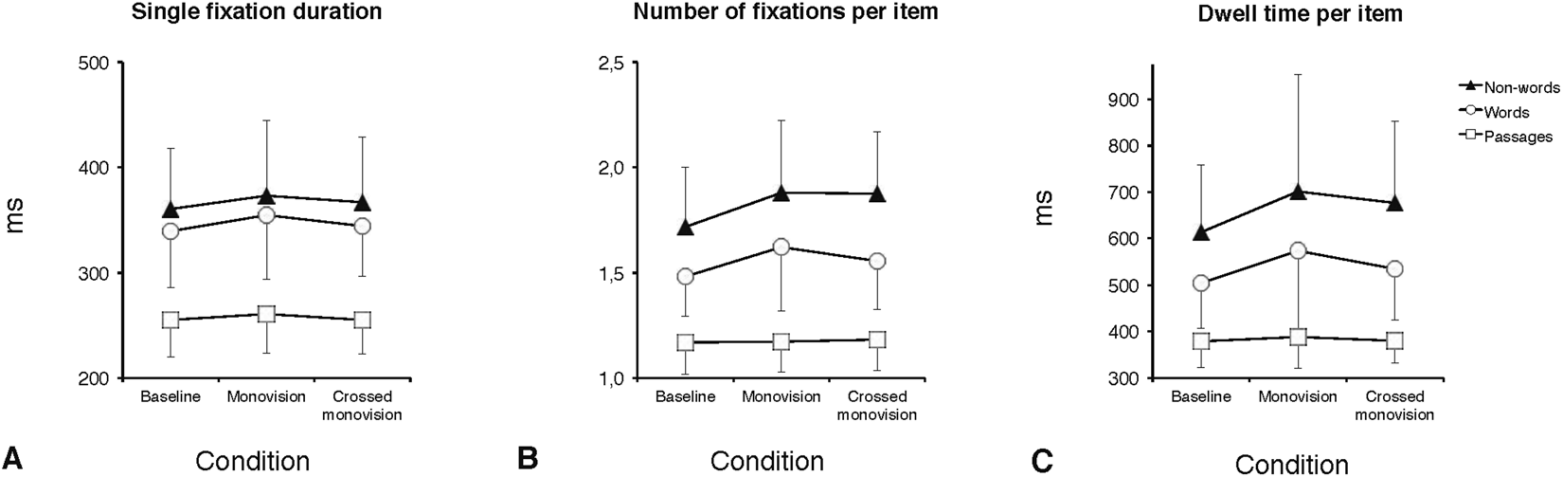
Group means for EM parameters: single fixation duration (**A**), number of fixations per item (**B**) and the dwell time per item (**C**). Data are separately presented for text passages, words, and non-words and as a function of the three experimental CL conditions (baseline, monovision, and crossed monovision). Error bars represent standard deviations.

**Figure 3:**
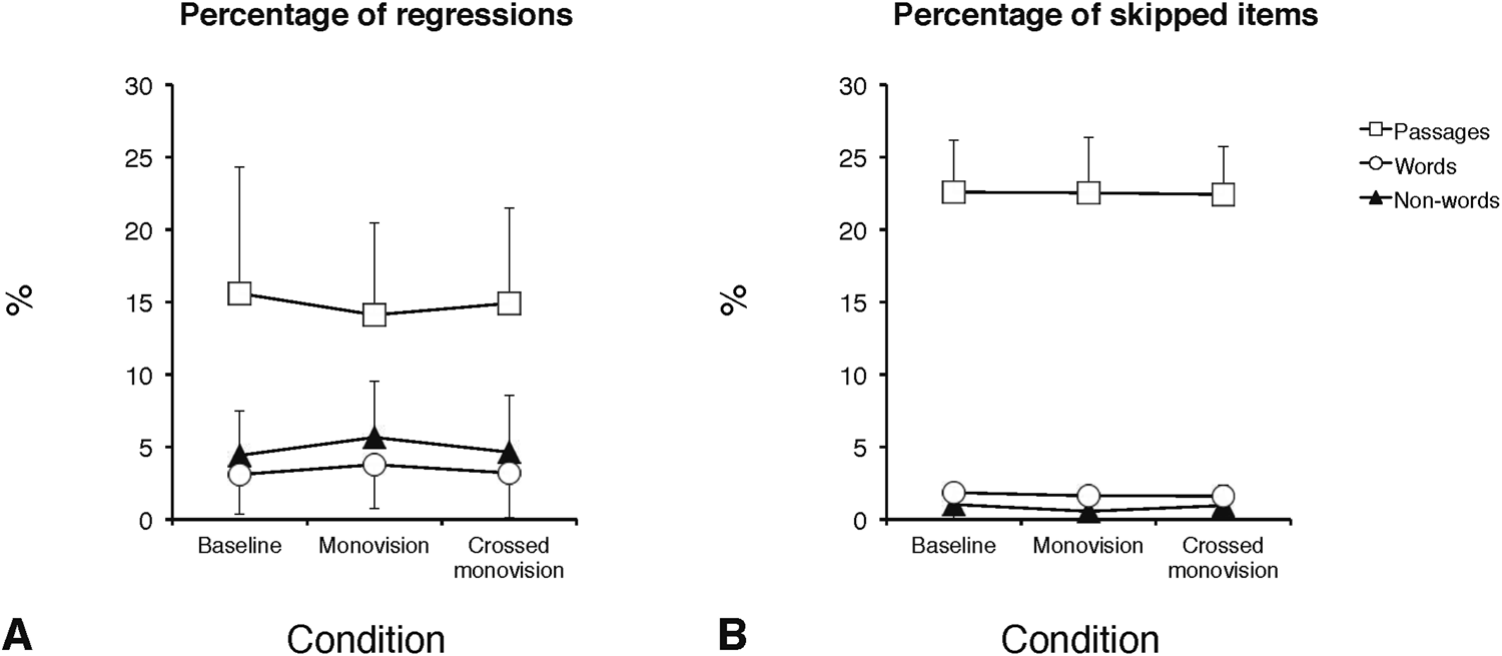
Group means for EM parameters: percentage of regressions (**A**) and of skipped items (**B**). Data are separately presented for text passages, words, and non-words and as a function of the three experimental CL conditions (baseline, monovision, and crossed monovision). Error bars represent standard deviations.

The effect of CL correction on passage reading was examined by one-way ANOVAs with CL condition (baseline, monovision, and crossed monovision) as within-subjects factor. ANOVAs were separately run for each EM (fixation duration, number of fixations per item, dwell time per item, percentage of regressions and percentage of skipped items) and behavioral (WPM for reading speed and percentage of errors for accuracy) parameter. Group means are presented in Table 3. ANOVAs showed that there was no significant effect of CL condition for any of the EM and behavioral parameters, indicating that wearing monovision CLs did not affect reading meaningful texts (Fig. S1 in the supplementary materials shows examples of spatial overlays of the fixations over portions of text passages in the baseline and the monovision conditions). In particular, WPM (Fig. 1A) did not differ across conditions (F_(2,28)_ = 3.5, p = 0.08) as well as the percentage of errors (Fig. 1B; F_(2,28)_ = 2.9, p = 0.07), which were very low, and changed by only 0.5% with monovision (that is, an average increase of less than 1 error on 185 words). All EM parameters were comparable across conditions: fixation duration (Fig. 2A; F_(2,28)_ = 2.0, p = 0.15); number of fixations per item (Fig. 2B; F_(2,28)_ < 1, p = 0.95); dwell time per item (Fig. 2C; F_(2,28)_ < 1, p = 0.39); percentage of regressions (Fig. 3A; F_(2,28)_ = 1.4, p = 0.26). Furthermore, the rate of skipped items was comparable across conditions (Fig. 3B; F_(2,28)_ < 1, p = 0.98), with percentages indicating that the participants were able to effectively skip short and long function words not only in the baseline, as expected for expert readers, but also in the monovision conditions.

**Table 3:** Group means (and standard deviations in parentheses) for eye movement and behavioral parameters measured while reading text passages, and word and non-word arrays, separately for the three CL conditions.

	Text Passages			Words			Non-Words		
	Baseline	Monovision	Crossed monovision	Baseline	Monovision	Crossed monovision	Baseline	Monovision	Crossed monovision
**Eye movement parameters**									
Fixation duration (ms)	255 (35)	261 (37)	255 (32)	339 (53)	355 (61)	344 (48)	360 (58)	373 (72)	367 (62)
Number of fixations per item	1.17 (0.2)	1.17 (0.1)	1.18 (0.1)	1.49 (0.2)	1.64 (0.3)	1.56 (0.2)	1.72 (0.3)	1.88 (0.3)	1.88 (0.3)
Dwell time per item (ms)	379 (57)	388 (67)	380 (48)	505 (97)	574 (169)	535 (111)	613 (144)	701 (253)	676 (176)
Percentage of regressions	15.6 (8.7)	14.1 (6.3)	14.9 (6.6)	3.1 (2.7)	3.8 (3.1)	3.2 (3.1)	4.4 (3.1)	5.7 (3.9)	4.6 (3.9)
Percentage of skipped items	22.6 (3.6)	22.5 (3.8)	22.4 (3.3)	1.9 (2.7)	1.6 (2.7)	1.6 (2.5)	1.0 (1.2)	0.6 (1.2)	1.0 (1.4)
**Behavioral parameters**									
Accuracy (% of errors)	1.8 (1.8)	2.5 (1.8)	1.7 (1.3)	1.8 (1.5)	3.1 (3.2)	2.8 (3.2)	5.1 (2.9)	8.5 (11.0)	6.6 (5.7)
Reading speed (WPM)	175 (23)	169 (21)	172 (20)	104 (17)	103 (26)	104 (18)	87 (19)	81 (19)	84 (24)

### Eye movement and behavioral parameters for the reading of word and non-word arrays

The effect of CL correction on word and non-word arrays reading was examined carrying out two-way ANOVAs with the three CL conditions (baseline, monovision, and crossed monovision) and type of stimulus (word, non-word) as within-subjects factors. ANOVAs were separately run for each EM and behavioral parameter as in the case of text passages. Group means are presented in Table 3, separately for word and non-word arrays. In the case of EM, a main effect of CL condition was present for the number of fixations per item (Fig. 2B; F_(2,28)_ = 6.91; p < 0.005) and for the dwell time per item (Fig. 2C; F_(2,28)_ = 5.02; p < 0.05). Separate post-hoc analyses (Tukey test) showed that monovision required more fixations (1.76; p < 0.005) and a longer dwell time (638 ms; p = 0.01) with respect to baseline (1.60 and 559 ms, for number of fixations and dwell time, respectively), and that crossed monovision required more fixations (1.72; p < 0.05) than baseline (Fig. S1 in the supplementary materials shows examples of spatial overlays of the fixations over portions of non-word arrays in the baseline and the monovision conditions). The effect of the CL condition was not significant for fixation duration (Fig. 2A; F_(2,28)_ = 2.0, p = 0.16) and percentage of regressions (Fig. 3B; F_(2,28)_ = 2.0, p = 0.15) as well as for the all behavioral parameters (WPM: F_(2,28)_ = 1.2, p = 0.31; percentage of errors: F_(2,28)_ = 1.6, p = 0.22; Fig. 1A,B, respectively). The main effect of stimulus type was significant for all EM parameters (except skipping rate), with longer (367 vs. 346 ms; F_(1,14)_ = 7.8, p < 0.05) and more numerous fixations (1.83 vs. 1.56; F_(1,14)_ = 32.0, p < 0.0001), longer dwell time (664 vs. 538 ms; F_(1,14)_ = 30.8, p < 0.0001), and more regressions (4.9 vs. 3.4; F_(1,14)_ = 14.6, p < 0.005) for non-words with respect to words, as expected when a serial processing to decode meaningless strings of letters is engaged. Indeed, non-words were read more slowly than words (84 vs. 104 WPM; F_(1,14)_ = 78.7, p < 0.0001) and produced a greater percentage of reading errors (6.8 vs. 2.6; F_(1,14)_ = 16.8, p < 0.001). The condition x stimulus type interaction was not significant for any EM or behavioral measure, indicating that the type of stimulus did not differentially affect performance as a function of CL condition. Finally, as expected for arrays of unrelated items, the skipping rate was very low, ranging from 0.6% to 2.0%, without any significant effect of CL condition (F_(2,28)_ < 1, p = 0.74) or stimulus type (F_(1,14)_ = 2.8, p = 0.12).

## Discussion

Consistent with previous research^9–14^, this study showed that monovision in a group of naïve presbyopes produced a complex pattern: on the one hand, monovision determined a drop of stereopsis, an increased level of suppression, a decrease of the VA at distance in the eye corrected for near and a slight worsening of reading acuity and CPS with respect to the balanced condition of both eyes corrected for near. On the other hand, there were a higher reading acuity and a smaller CPS than those obtained with the subjective refraction at distance, meaning that the presbyope is now able to read small typographical characters thanks to the CL that corrects for near vision in one eye despite the other eye wearing a CL correction for distance.

Furthermore, examining for the first time EM traces during reading under monovision this study did not find any significant effect of the use of monovision on the reading of text passages with respect to the use of a balanced condition with both eyes corrected for near. This finding is important since EMs are presumably quite sensitive in detecting even small effects on visual scanning (see below for further comments in the case of arrays of unrelated stimuli). The efficiency of reading text passages in monovision conditions was confirmed by the observation that word skipping, a typical feature of experienced reading^20^, was unaffected by monovision.

Some selective influence of monovision was detected only in the case of arrays of unrelated stimuli such as the words or non-words. In particular, the number of fixations increased in monovision and the dwell time was longer. Since fixation duration did not change, it seems that the critical change is in the scanning process, represented by the number of fixations (which contribute to the determination of dwell time). Therefore, it is not the temporal sampling itself to be affected but the spatial sampling: indeed, a denser spatial sampling of text is revealed by the presence of more forward fixations in the direction of reading, but not regressions, since the percentage of regressive saccades back to previous items or to the left part of a same item did not differ. Finally, the effect of the monovision conditions did not depend on the lexical value of the stimulus; in fact, no significant interaction between the CL condition and the type of stimuli (words/non-words) emerged. This indicates that the effect that monovision exerted on reading was at an early (presumably pre-lexical) level of processing.

Overall, examination allowed detecting some influence of monovision on EM reading parameters (but not on behavioral indexes) in the case of stimuli devoid of meaning (due to the absence of contextual information). Notably, the sensitivity of the EM in detecting even small differences in visual scanning suggests that the absence of monovision influences observed in the case of text passages cannot be easily ascribed to the insensitivity of the measures considered.

Since this study has tried to explore for the first time the effect of monovision on reading EM several questions remain open for further investigation.

Firstly, in this study, only one level of power addition at near (+1.75 diopters) was used to induce a relevant stress to be detected by EM measures. Clinicians are aware that the level of power addition at near can be critical to monovision adaptation and success^6^ because the higher the add power the stronger the binocular stress induced. It is possible that levels of addition higher than +1.75 diopters may affect reading EM differently from the present results that therefore cannot be extended to other powers of correction. For example, higher levels of add, that are typically used by older people (e.g., over 60 years old), could be studied; nevertheless, monovision is not optimal with near addition powers of more than +2.00 diopters^4,7^. Further research is needed to understand the effect of levels of addition powers different to that used in this study.

Secondly, it would be interesting to verify by follow-up studies the conditions that may determine the success or failure of monovision, examining subjective and objective aspects potentially responsible for either the continuation or drop out of this method of correction. Assessment and EM data could be retrospectively examined to discriminate between people that are going to be successful from people destined to fail when using monovision as habitual correction of presbyopia. In addition, activities both at near and at far distance may be jointly examined in the same participants that successfully adopted monovision as a long-term correction. This is an important issue, since reduction of visual acuity at distance and problems regarding contrast sensitivity and binocular vision may potentially affect activities at far distance such as driving.

Finally, a potentially interesting aspect to explore is the effect of adaptation to monovision. The present study was carried out on subjects completely naïve to monovision but if monovision CLs were worn for a longer period, the effect on EMs are likely to change over time. For example, Sheedy *et al*.^21^ found that several everyday tasks were performed more accurately after a period of adaptation to monovision.

Overall, the reading of passages with monovision CLs is effective (at least for short periods of time), despite the drop of some visual functions (binocular fusion and stereopsis) shown by the visual assessment. In particular, not only behavioral parameters (i.e., reading speed and accuracy) but also sensitive measures, such as EM parameters, were not affected. Monovision altered the EM reading pattern only in the case of arrays of unrelated items or when items must be scanned in a serial way because the correspondence with the lexicon is missing. In these cases, the reading scanning was characterized by an increase in the number of fixations that contributed to a prolonged total fixation time of an item. Nevertheless, neither accuracy nor reading times got significantly worse, suggesting that at least for short periods of time reading is resilient to the visual stress due to an induced anisometropia. This effect of monovision on EM but not behavioral reading (speed and accuracy) parameters is consistent with the result of an adaptation of brain activity recently found in a study of our group carried out examining visual evoked potentials (VEPs) in a group of presbyopes wearing CLs in a monovision condition^22^. Taken together, these studies suggest that the visual system can effectively respond to the imbalance of the visual correction by adopting modifications that are seamless at the behavioral level. To conclude, the experimental evidence from the present study demonstrate that reading of texts is not negatively affected by monovision. Thus, monovision remains a suitable option for presbyopia correction, at least for the levels of near additional power (+1.75 diopters) used in this study.

## Methods

The study was based on a repeated measures design examining reading time and accuracy (behavioral parameters) and several EM parameters during text reading at near distance under three CLs conditions. The study was conducted in keeping with the Declaration of Helsinki and was approved by the Ethics Committee of Fondazione Santa Lucia of Rome, Italy (rec. no. CE/PROG.602). After a detailed explanation of the procedures, informed consent was obtained from all participants to participate in the study.

### Participants

Fifteen healthy presbyopic Italian native-speaking volunteers (8 males and 7 females; mean age 48.9 ± 3.1 years; age range 45–57 years). The inclusion criteria were the absence of any ocular pathology, being a presbyope, naïve to monovision CLs, having a good binocular vision and good stereopsis, and having a BCVA not lower than 0.1 logarithms of the minimum angle of resolution (logMAR) with a difference between the two eyes lower than 0.1 logMAR.

### Preliminary visual assessment

The visual assessment was conducted by a licensed optometrist. Ophthalmoscopy and slit-lamp examination were carried out to detect any ocular anomaly. Ocular motility was examined by using a H pattern test. Dissociated phorias (either at distance and near through an alternating cover test and prism bar), fusional reserves (at distance with prism bar), and near point of convergence were assessed to detect any binocular vision anomaly. Eye sighting dominance was determined by the Hole-in-the-Card test. Non-cycloplegic subjective refraction at distance was carried out monocularly by a phoropter procedure with a final equalization via dissociated testing^23^ and the mean spherical equivalent (MSE) was determined. Addition for near was firstly determined according to the expected age procedure^24^ and then adjusted subjectively to obtain the final addition^25^.

High contrast monocular BCVA was measured at a distance of 5 meters using Sloan letters on a Bailey-Lovie chart displayed on an LCD optotype system (CSO; Florence, Italy)^26^. Donder’s push-up method (RAF rule) was used to measure the amplitudes of accommodation^25^.

Stereoacuity, fixation disparity, suppression, reading acuity and critical print size (CPS) were measured with the subject wearing their near refractive correction. The Borish Vectographic Nearpoint card II (Stereo Optical Company, Chicago, IL USA) was presented with the correction at near placed on both eyes to measure the stereoacuity threshold and check for the possible presence of near horizontal fixation disparity at the 40 cm distance. If fixation disparity was present, associated phoria (in prism diopters) was determined. The level of central suppression was measured by the modified Borish test^27^ on a scale from 0 to 5 (0 = no reported suppression; 5 = constant monocular suppression of one eye) with the correction at near in both eyes. A subjective measure of the impact of presbyopia without the use of any correction at near was obtained through the validated Italian version^28^ of the Near Activity Vision Questionnaire (NAVQ)^29^, which measures the subjective satisfaction of the quality of vision at near.

Reading acuity and critical print size (CPS) were measured binocularly with the subjective refraction at distance and with the correction at near (in randomized order) whilst participants read randomized test charts of the standardized Italian version of the Radner test at 40 cm^30^.

After having determined the CL power for the experimental conditions (see below), each participant was fitted for the first time with monovision. Distance VA for the eye fitted for near vision, stereopsis, and central suppression were measured. Reading acuity and CPS were also measured in monovision using the Radner chart at 40 cm.

### Experimental design and CL manipulations

All participants wore CLs with visual corrections according to the following three experimental conditions:

- Baseline: both eyes were corrected for near vision with single vision CLs equal to the MSE plus a near additional power of +1.75 diopters;
- Monovision (i.e., the conventional monovision correction): Dominant eye was fitted a single vision CL with a power coincident to the MSE; non-dominant eye was fitted with a single vision CL with a power equal to the MSE plus an addition of +1.75 diopters;
- Crossed Monovision: Dominant eye was fitted a single vision CL with a power equal to the MSE plus an addition of +1.75 diopters; non-dominant eye was fitted with a single vision CL with a power equal to the MSE.

Conditions were presented in a randomized sequence across participants.

The dioptrical difference between the distance corrected eye and the near corrected eye was maintained at a level of +1.75 diopters in the two conditions of monovision (conventional and crossed) for all participants in order to maintain an equal level of stress between the two eyes across participants during the anisometropic conditions.

All CLs were daily disposable (Proclear 1-day, Cooper Vision, Victor, NY, USA), Omafilcon A, water content of 60%, back optic zone radius 8.7 mm, total diameter 14.2 mm and Dk/t 28.

### Eye movement recordings: apparatus, stimuli, and procedure

EM were recorded in binocular vision via an SR Research Ltd. Eye Link 1000 eye tracker (SR Research Ltd., Mississauga, Ontario, Canada) sampling at 1000 Hz, with a spatial resolution of 0.05 deg. Synchronous to EM traces, the reading aloud was digitally recorded using a microphone interfaced to the eye tracker.

Stimuli were displayed at a viewing distance of 40 cm on a screen (resolution: 1024 × 768 pixels; refresh rate: 85 Hertz) interfaced to the eye tracker. All stimuli were written in Courier font, with black letters on a white background. Average center-to-center letter distance subtended 0.3 deg.

Text passages subtended a visual angle of 17.0 × 8.0 degrees and were horizontally displayed at the center of the screen and at 12.1 degrees from the top edge of the screen. Twelve passages were taken from the tale *Aesop’s Fables*; each passage comprised 10 lines of text. The words of the central 8 lines (excluding the first and last word of each line) served as targets for EM analyses; the remaining items were fillers. Passages were matched for the number of target content words, for content words frequency of use, and for word length in letters (on average 61.5 ± 3.3 target words/trial; average word length: 4.7 ± 2.5 letters). To avoid global appearance differences among trials, the whole content of the passages in terms of the total number of words and characters was matched.

Word and non-word arrays subtended a visual angle of 15.9 × 3.7 degrees and were displayed at the center of the screen and 12.1 degrees from the top edge of the screen. A single trial was comprised of 45 items distributed in 5 lines. For each trial, there were 18 central 5-letter items (six per line, from the second to the fourth line) that served as targets for EM analyses; the remaining items (the first and last lines of the text, and the first item and the last two of the three central lines) varied in length and were considered as fillers. All items were set up according to an identical spatial arrangement of lengths across trials so that the global appearance of the 24 trials was the same. Regularity of disposition in columns within a trial was avoided by insertion of fillers of different length at the edges of the text. Target word frequency was matched across the 12 word-trials. Non-words were derived from words by changing one (or two) letter(s), or three letters for some 6–7-letter fillers. Non-words order within a trial differed from the order of the word-trial from which they were derived, but both trials were assigned to the same CL condition and recorded eye.

The traces from the left and right eye were recorded in all participants. The ambient illumination level was constant across recordings (room lighting of 230 lux). The participant sat comfortably and maintained a steady position. Head movements were avoided by using a headrest; the chin was left free for ease of articulation. A nine-point calibration was run before showing each text. The targets appeared in random positions; the participant fixated each point steadily. The calibration was repeated before each experimental condition, separately before left or right eye recordings. Immediately after calibration, a cross appeared in the upper-left quadrant of the screen (1.4° to the left of the first letter of the text), serving as initial fixation target. The offset of the cross and the simultaneous onset of the display containing the text was automatically triggered when the participant steadily fixated the cross for at least 150 ms. Participants were asked to read aloud as quickly and accurately as possible. Texts were removed after the end of the last word uttered.

There were 12 trials (4 passages, 4 word arrays and 4 non-words arrays) for the baseline, 12 for the monovision, and 12 for the crossed monovision conditions for a total of 36 different text trials. For each set of four trials of the same type of stimulus (i.e., either passages, words, or non-words), two trials were used for the left and two for right eye recordings (under binocular vision).

Approximately 10 minutes before starting the EM recordings the pair of CLs for the specific condition was fitted in order to reach a good comfort and avoid the presence of reflex tearing and/or an excessive rate of blinks. The whole recording session lasted approx. 45 minutes. A practice run based on three trials (one screen per stimulus type) was administered before the presentation of the first set of experimental trials to familiarize the participant with the calibration procedure and the reading tasks. The three CL conditions were administered as follows: after the training, the first condition started; at the end, there was an interval of 10 minutes allowing CL substitution and adaptation to the novel vision condition. At the end of the second condition, there was another 10-minute interval allowing CL change and a new adaptation. The order of CL conditions, then of recorded eye, and of stimulus type, was randomized across participants.

### Eye movement and audio recording processing

EM data were processed via EyeLink Data Viewer software (SR Research Ltd., Mississauga, Ontario, Canada). All parameters were measured based on reliable portions of the eye traces, i.e., recordings not containing artefacts such as eye-blinking (Fig. S2 in the supplementary materials shows an example of the temporal graph illustrating a portion on the eye movement showing reliable parts of the trace as well as artifacts). These portions were automatically signaled by the recording system and could be discarded (along with the stimulus they corresponded to); additionally, the traces were also manually checked by the experimenter looking for any abrupt loss of eye trace across the eye movement pattern corresponding to the visual region containing the text to be read. Average fixation duration, number of fixations per item, dwell time per item (i.e., the sum of the durations of all fixations on a same word or non-word), and percentage of regressions (i.e., backward saccades to previously fixated text) were computed on the portion of text containing the target items. Furthermore, the proportion of items that were skipped (i.e., did not receive any fixation) by the reader in each text was examined. Skipping short and long function words (such as articles and adverbs) in meaningful texts (such as the text passages used in the present study) is a typical sign of flexibility shown by expert readers^20^ and characterizes fluent and effective reading.

Audio recordings served for measuring behavioral parameters (reading speed in WPM, and accuracy in terms of percentage of errors) on the portion of text containing the target items, and were processed via Audacity 2.1.1 software using a mixed criterion of visual inspection of the waveform image and listening to the audio track.

Repeated measures ANOVAs were performed separately for every parameter using the SPSS software. Left and right eye recordings were pooled together to reduce the number of factors and simplify the presentation of ANOVAs results (preliminary analyses indicated no effect or interaction with an “eye” factor).

## Electronic supplementary material

Supplementary Information
