# Supplementary material for: Pattern of reading eye movements during monovision contact lens wear in presbyopes
Source: Sci Rep. 2018 Oct 22;8:15574. doi: 10.1038/s41598-018-33934-6 (PMC6197269; doi:10.1038/s41598-018-33934-6)

Supplementary materials

Pattern of reading eye movements during monovision contact lens wear in presbyopes

Fabrizio Zeri[1,2,*], Shehzad A. Naroo[1], Pierluigi Zoccolotti[3,4], Maria De Luca[3]

[1] Ophthalmic Research Group. School of Life and Health Sciences, Aston University, Birmingham, B4 7ET, United Kingdom
[2]University of Milano Bicocca, Department of Materials Science, 20125 Milan, Italy
[3] Neuropsychology Unit, IRCCS Fondazione Santa Lucia, Rome, 00179, Italy
[4] Department of Psychology, Sapienza University, Rome, 00176, Italy
* f.zeri@aston.ac.uk

Figure S1

Examples of portions of eye tracking elaborations. In these spatial overlays, the circles represent the localizations of eye fixations over the text separately for the baseline and monovision conditions, for the passages and the non-word lists. The size of the circles is proportional to fixation duration. Note the denser distribution of fixations for the list of unrelated items (non-words) in the monovision condition as compared to the baseline. No difference is appreciable comparing baseline and monovision in the case of passage reading.

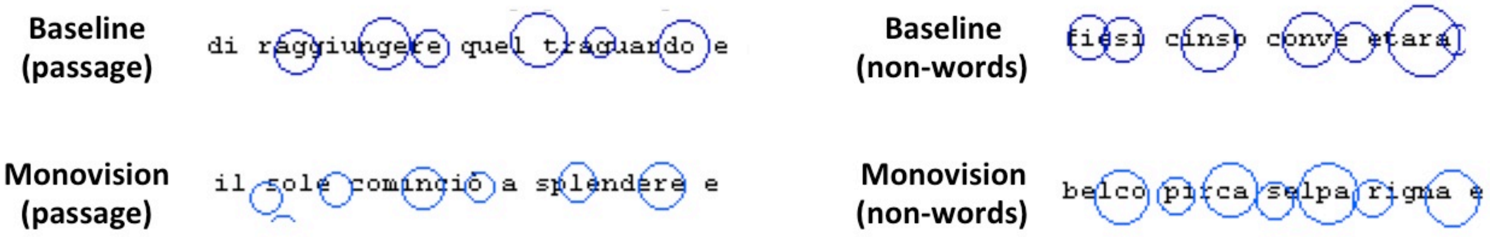

Figure S2

Temporal graph of the EM traces showing the typical staircase pattern observed during the reading of a text passage. Time (ms) is plotted on the abscissa (x-axis) and space (pixels) on the ordinate (y-axis). For the ordinate, the top portion corresponds to the right and the lower portion to the left side of the presented text. The vertical orange bars represent losses of signal that are automatically detected by the recording system as unreliable portions of the trace (often due eye blinks as in the bar to the right). Fixations and text items (either a word or a non-word in the case of the present study) involved in such artefacts were not included in the analysis.

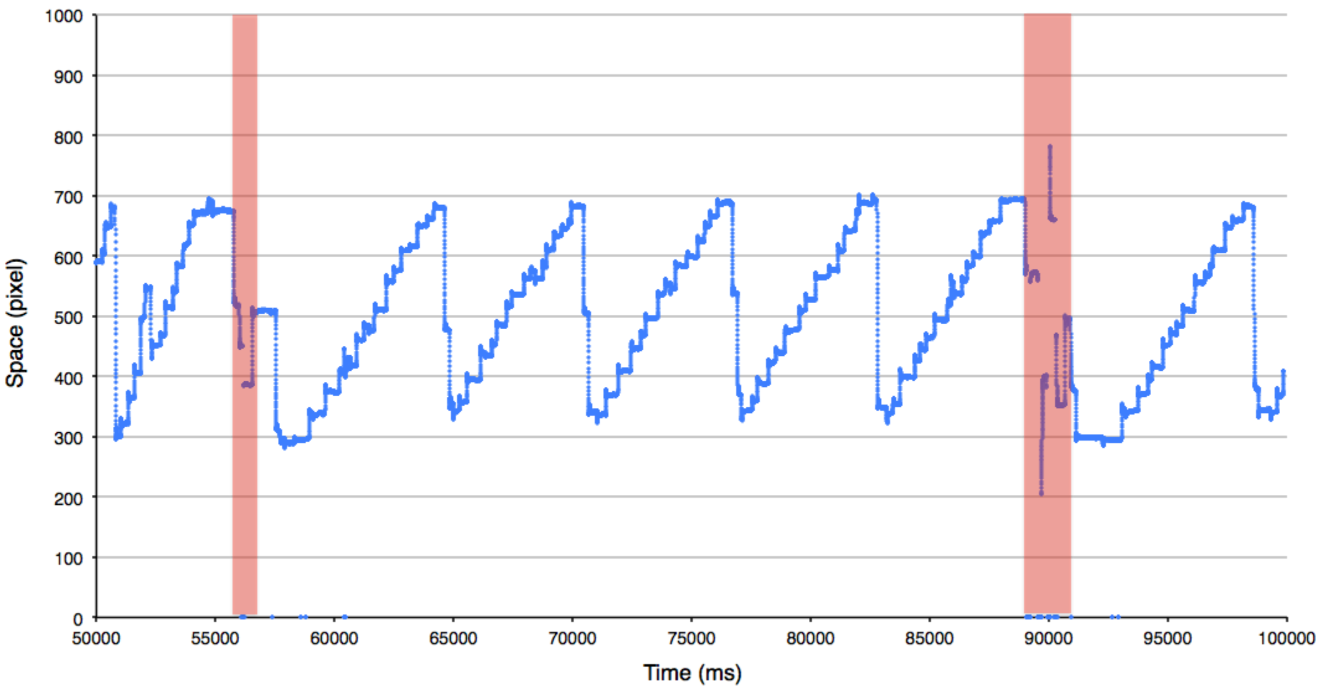

Supplement: Supplementary file 1 — Supplementary Information [file 41598_2018_33934_MOESM1_ESM.pdf]
